# Using Motivational Interviewing to Increase HIV PrEP Initiation and Adherence: a Scoping Review

**DOI:** 10.1007/s11121-023-01554-w

**Published:** 2023-05-30

**Authors:** Derek T. Dangerfield, Gerrin Davis, Vinciya Pandian, Janeane N. Anderson

**Affiliations:** 1 Department of Prevention and Community Health, George Washington University Milken Institute School of Public Health, 950 New Hampshire Ave NW, Washington, DC 20052 USA; 2 Johns Hopkins School of Nursing, Baltimore, MD USA; 3 College of Nursing, University of Tennessee Health Science Center, Memphis, TN USA

**Keywords:** Prevention, Brief intervention, Men who have sex with men, Women, Sexual health

## Abstract

Despite evidence that pre-exposure prophylaxis (PrEP) reduces HIV risk, initiation and adherence remain low among vulnerable communities. Motivational interviewing (MI) can improve HIV prevention behaviors. However, limited research identifies how MI impacts PrEP uptake and adherence. This scoping review examines essential components of MI-based interventions that aimed to improve PrEP use, including the number and duration of sessions, counselor characteristics, and interview content. We searched four databases, PubMed, CINAHL Plus, Embase, and Web of Science, and reviewed 379 articles. Studies were considered if they (a) were published between 2012 and 2023, (b) used MI independently or part of a multi-component intervention strategy, and (c) focused on improving PrEP initiation or adherence. Seven articles met inclusion criteria. Regarding intervention components, the number of MI sessions varied and duration ranged between 15 and 60 min. MI counselors varied in credentialing and demographic characteristics. MI content included PrEP education, identifying initiation and adherence barriers, and strategizing ways to overcome barriers. MI is an important component of interventions that aim to improve PrEP initiation and adherence. However, the variability and limited details across studies hinder our ability to assess MI efficacy on PrEP initiation and adherence or replicate these approaches in future interventions.

## Introduction

Increased HIV pre-exposure prophylaxis (PrEP) initiation and adherence can help end the epidemic because it substantially reduces acquisition risk among vulnerable individuals (Anderson et al., 2012; Fauci et al., 2019; Mayer et al., 2020; Centers for Disease Control and Prevention [CDC], 2021). Specifically, PrEP adherence can reduce HIV acquisition risk by 99% when taken as prescribed (CDC, 2021). However, PrEP use remains suboptimal among individuals at greatest risk for HIV due to multi-level factors such as limited healthcare access, medication stigma, discomfort discussing sexual risk behaviors with clinicians, and low perceived HIV risk (Cahill et al., 2017; D’Angelo et al., 2020; Felsher et al., 2018). Disparities in PrEP uptake based on age, race, gender, and sexual orientation also exist (Kanny et al., 2019). Scalable and cost-effective interventions such as motivational interviewing (MI) (Bonacci & Holtgrave, 2017; Kelly, 2019) could circumvent barriers, reduce disparities, and improve PrEP initiation and adherence.

MI is a collaborative communication approach in which a counselor partners with clients to activate their motivations and resources to change unhealthy behaviors (Miller & Rollnick, 2012; Rollnick & Miller, 1995; Rollnick et al., 2010). MI facilitates progress through the transtheoretical model of change (Prochaska & DiClemente, 1982) and consists of the following core concepts: (1) engaging clients in collaboration with the counselor, (2) focusing on client goals, (3) evoking client motivations and barriers to behavior change, and (4) planning next steps toward the desired behavior(s) (Levensky et al., 2007; Miller & Rollnick, 2012). MI typically consists of one or two sessions ranging between 10 and 120 min (Hettema et al., 2005; Rubak et al., 2005) and can be conducted in a variety of settings such as clinics, community-based organizations, and via telephone (Hettema et al., 2005; Kahler et al., 2020; VanBuskirk & Wetherell, 2014). MI counselors can be trained paraprofessionals or clinicians who use open-ended interviewing and reflective listening to identify client ambivalence and barriers to behavior change (Naar-King et al., 2009; Palacio et al., 2016; Rollnick & Miller, 1995). MI approaches are often used in health promotion because of their cost-effectiveness, session brevity, adaptability, and use of client interests for behavior change. MI began with addiction counseling and has been applied as an intervention strategy globally to support effective behavior change for many health behaviors (Rubak et al., 2005), including dieting (Stallings & Schneider, 2018), exercise (Barnes & Cassidy, 2018), violence prevention (Soleymani et al., 2018), and medication adherence for chronic conditions (Ekong & Kavookjian, 2016).

MI can also be used to promote HIV prevention behaviors such as condom use, HIV testing, and PrEP use. Some biobehavioral HIV prevention interventions utilize “MI-consistent” or “MI-based” interviews because of its benefits (Carcone et al., 2022; MacDonell et al., 2022; Moitra et al., 2020). These interventions are useful to reduce barriers such as stigma and perceived HIV risk and could be effective at improving initiation and adherence. Research in other prevention domains has examined the influence of MI counselors’ personal characteristics, professional credentialing, and/or training on intervention efficacy (Gaume et al., 2009, 2014, 2016), highlighting the need for this inquiry among HIV prevention interventions. However, limited information is available regarding the specific components of MI-based interventions that aid in improving PrEP initiation and adherence. Few studies have clarified the credentials of interventionists, the intervention setting, discussion length, client goals, and conversation content in PrEP initiation and adherence interventions. Therefore, HIV prevention scientists’ understanding of how MI impacts PrEP use is limited. Additionally, optimizing and potentially standardizing MI-based interventions to improve PrEP initiation and adherence among individuals at greatest risk for HIV acquisition is challenging without details of effective key components.

The purpose of this scoping review is to examine the use of MI interventions that aimed to increase PrEP initiation and adherence and identify their components. Identifying the components of MI-based interventions (i.e., the counselor, setting, session duration, and content) is necessary to clarify the meaning of “MI-consistent” or “MI-based” approaches for implementing research protocols systematically. Inferences regarding the effects of MI-based interventions in PrEP interventions are difficult to make because details of MI components in the strategy are lacking. Replicating these PrEP promotion strategies is also difficult because details of their MI components are typically missing. Moreover, a better understanding of intervention components in PrEP interventions is needed to identify how contemporary strategies differ or deviate from original MI frameworks. Identifying components of MI-based protocols, including the counselor, number and duration of sessions, and content, is needed to establish a consistent MI-based approach among PrEP interventions and better evaluate the impact of MI on PrEP initiation and adherence. Findings will allow HIV prevention scientists (and clinicians) to implement more effective, reliable, and scalable interventions to increase PrEP initiation and adherence among vulnerable communities.

## Methods

### Search Strategy

**Fig. 1:**
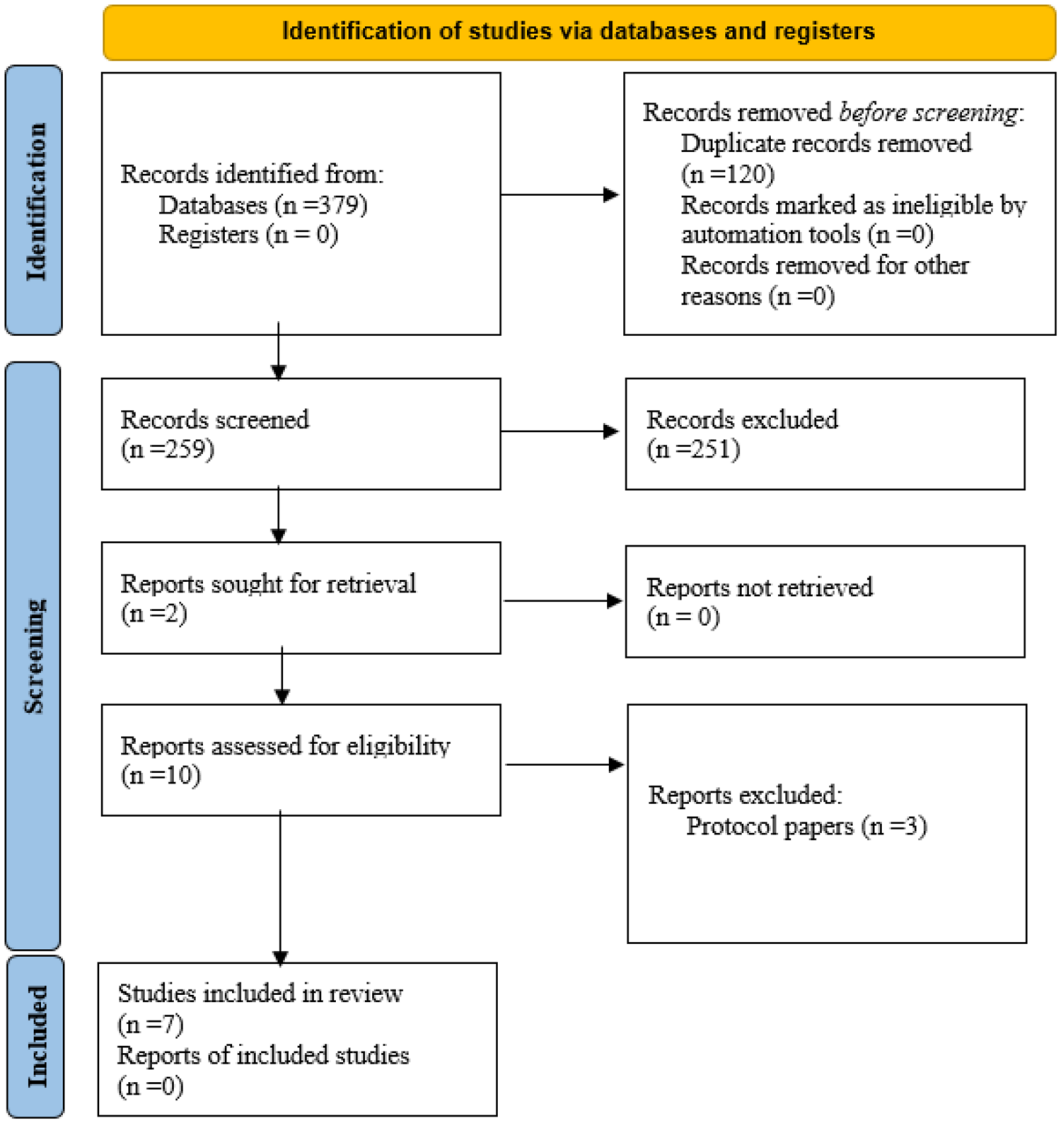
PRISMA flow diagram (Page et al., 2021)

A targeted literature search guided by the Preferred Reporting Items for Systematic Reviews and Meta-Analyses (PRISMA) checklist (Fig. 1) (Moher et al., 2009) was conducted between October 2020 and March 2023. To identify a range of relevant epidemiological, public health, and social science literature, we searched four databases: PubMed, Cumulative Index for Nursing and Allied Health Literature Plus, PsycINFO, and Embase. The literature search was conducted using the following search terms across all databases: “Human Immunodeficiency Virus OR HIV,” “HIV prevention,” “Pre-exposure Prophylaxis OR PrEP,” “Emtricitabine-Tenofovir Disoproxil Fumarate Drug Combination OR Truvada,” “Motivational Interviewing OR MI,” “Brief Intervention,” and “Descovy OR Emtricitabine/tenofovir alafenamide.”

### Study Selection

Studies were considered for inclusion if they (1) were published between 2012 and 2023, (2) used MI as an independent intervention, or (3) used MI as part of a multi-component strategy to improve PrEP initiation or adherence. Studies among all populations and in global settings were considered in order to identify and review the full scope of MI-related interventions among diverse groups in different cultural and geographic contexts. Dissertations, books, editorials, letters, and commentaries were excluded.

This strategy yielded a total of 379 studies after duplicates were removed. After initial review of titles and abstracts, ten full-text articles were assessed for inclusion, seven of which are included in this review. Three articles were excluded because they described intervention protocols without providing details regarding intervention effects on study outcomes (Fig. 1) (Page et al., 2021).

Data abstracted from the seven studies include the study sample, study design, MI setting, motivational interviewer, duration of sessions, theoretical framework, and MI content (Table 1). We used the Johns Hopkins Evidence-Based Practice Quality Guide (Dang & Dearholt, 2018) to identify the level of evidence and appraise the quality of the included studies in Table 2.

**Table 1:** Characteristics of studies using motivational interviewing to increase PrEP initiation and/or adherence

**First author (year)**	**Sample**	**Study design**	**Theoretical framework**	**MI setting**	**Interviewer**	**MI session** **Duration**	**MI content**	**Intervention effect**
Chan et al. (2021)	86 SMM	RCT	MI theory	STI clinic and telephone USA	State-certified STI clinic counselors	2 sessions 15–20 min	Session 1: Build rapport, PrEP education, discuss HIV risk behaviors, identify and address barriers, PrEP pros and cons Session 2: Reinforce Session 1 and construct an action plan	Yes
Dale (2020)	10 Black women	Open pilot trial	Information, motivation, and behavioral skills model	Unspecified clinical setting Miami, FL, USA	Black female clinician	2 sessions plus 1-month follow-up	Session 1: PrEP information, enhancing motivation, work through ambivalence Session 2: Session 1 content plus questions regarding PrEP and potential access since Session 1	No
Mayer et al. (2017)	50 SMM adults	RCT	Cognitive Behavior Theory	Primary care clinic Boston, MA, USA	Nurse counselors	4 sessions plus 2 booster sessions (50 min)	Session 1: PrEP education, MI exercise, exploring adherence schedule Session 2: Adherence check-in, PrEP experience, problem solving Session 3: Adherence check-in, identifying sexual risk behaviors, health behavior/HIV transmission education Session 4: PrEP adherence goals and plans for PrEP maintenance Booster sessions: PrEP adherence	Yes
Moitra et al. (2020)	19 SMM adults	Uncontrolled pilot trial MI intervention	Self-regulation theory	Publicly funded STD clinic Rhode Island, USA	Unspecified	2 sessions 15–25 min	Unspecified	No
Morris et al. (2022)	255 transgender and non-binary adults	RCT	Theory of planned behavior	Telephone sessions, Southern California, USA	Research coordinators	Median 0–7 sessions, unspecified duration	Barriers and facilitators to adherence and adherence techniques	No
Psaros et al. (2014)	168 HIV-negative men & women in serodiscordant relationships	Placebo-controlled efficacy trial	Cognitive behavioral theory	Clinical research sites Uganda	Lay counselors	Varied, median 10 min (IQR 5, 16) 40 min (IQR 25, 30)	Reviewing pros and cons of achieving high levels of PrEP adherence. Resolving ambivalence about adherence, moving participants to a higher level of readiness to adhere	Yes
Teixeira da Silva et al. (2021)	136 Black SMM and transwomen	RCT	Information, motivation, behavioral skills model	Unspecific research site Chicago, USA	Social work interventionist	60 min, 4 booster sessions	HIV and PrEP information, motivation to reduce HIV risk and engage in PrEP care, HIV risk reduction strategies, planning to enact change	Yes

*RCT* randomized controlled trial, *SMM* sexual minority men, *IQR* interquartile range

**Table 2:** Summary of level and quality of evidence

**First author (year)**	**Observable measures**	**Intervention effects**	**Study limitations**	**Level & quality of evidence**
Chan et al. (2021)	Scheduling a PrEP appointment with a clinician Attending clinical appointment Accepting PrEP prescription via self-report	52.3% clinical appointment attendance and PrEP prescription for intervention group vs 27.9% in control group	Small sample of mostly white and educated SMM Self-reported PrEP prescription acceptance	**Level I** **Grade B** Good quality
Dale (2020)	PrEP use motivation and readiness PrEP knowledge PrEP uptake via self-report PrEP adherence via self-report	30% PrEP uptake	Small sample size MI counselor credentials unavailable Conclusions regarding intervention impact on PrEP initiation/adherence cannot be drawn	**Level III** **Grade C** Low quality
Mayer et al. (2017)	PrEP adherence at 6 months via Wisepill Tenofovir plasma levels Sexual risk behaviors	PrEP adherence high in both study groups, not statistically different	Small sample of mostly high risk, white, educated SMM Conclusions regarding intervention impact on PrEP adherence cannot be determined	**Level I** **Grade B** Good quality
Moitra et al. (2020)	PrEP awareness and use Intervention acceptability	High intervention acceptability 37% PrEP prescription after intervention	Small sample size Information regarding intervention content not available Conclusions regarding intervention impact on PrEP adherence cannot be determined	**Level III** **Grade C** Low quality
Morris et al. (2022)	PrEP adherence via dried blood spot concentrations ≥ 1246 fmol/punch Self-reported adherence	No statistically significant difference between the intervention and control arms (34.7% vs 38.3%; *P* = 0.60) or for the dried blood spot concentrations	Details regarding the MI content not available Details regarding MI counselors limited Conclusions regarding how intervention impacted PrEP adherence cannot be determined	**Level I** **Grade B** Good quality
Psaros et al. (2014)	PrEP adherence via pill count and micro-electromechanical system	8% mean increase in PrEP adherence post-intervention ≥ 80% PrEP adherence after first session	MI only used in the first session of intervention Large number and range of sessions Second session was couples-based Participants received different number of sessions	**Level I** **Grade B** Good quality
Teixeira da Silva et al. (2021)	linkage to PrEP care where PrEP was discussed with a clinician (regardless of initiation)	Greater proportion of intervention participants were linked to PrEP care within the linkage window (24% vs. 11%; *p* = 0.04) A significantly greater proportion of intervention participants initiated PrEP at 3-month follow-up (11% vs 25%; *p* = 0.05)	Small sample of transwomen limits ability to specify intervention effects	**Level 1** **Grade B** Good quality

## Results

### Study Population and Sample

Six of the seven studies were conducted in the USA; one study was conducted in Uganda. Four studies investigated MI with sexual minority men (SMM) (Chan et al., 2021; Mayer et al., 2017; Moitra et al., 2020; Teixeira da Silva et al., 2021). Four of the studies included non-SMM individuals, specifically U.S. Black women (Dale, 2020), transgender adults (Morris et al., 2022; Teixeira da Silva et al., 2021), and Ugandan men and women in serodiscordant relationships (Psaros et al., 2014). The sample size of these studies ranged from 10 to 255 (Table 1).

### Theoretical Frameworks

All studies utilized theoretical frameworks that guided the intervention (MI approach). Specifically, two studies were guided by cognitive behavior theory (Mayer et al., 2017; Psaros et al., 2014), two were guided by the Information, Motivation, and Behavioral skills (IMB) model (Dale, 2020; Teixeira da Silva et al., 2021), one was guided by the Theory of Planned Behavior (Morris et al., 2022), and one was guided by Self-Regulation Theory, a framework for understanding motivation and goal attainment (Moitra et al., 2020). Chan et al. (2021) reported using unspecified “MI theory” in an intervention among SMM.

### MI Intervention Setting

Six of the studies reported that their interventions were implemented in clinical settings, including sexually transmitted infection (STI) clinics (Chan et al., 2021; Moitra et al., 2020), primary care setting (Mayer et al., 2017), and unspecified clinical research sites (Dale, 2020; Psaros et al., 2014; Teixeira da Silva et al., 2021). One study included telephone-based MI sessions (Morris et al., 2022). Although most studies reported that the MI sessions were conducted in a traditional face-to-face format, three studies reported using telephone and/or video-based communication technology to deliver the intervention in a digital format (Chan et al., 2021; Morris et al., 2022; Teixeira da Silva et al., 2021).

### Motivational Interviewers

Three studies reported that clinicians conducted MI (Chan et al., 2021; Dale, 2020; Mayer et al., 2017). In two of these studies, state-certified STI clinic counselors and nurse counselors served as motivational interviewers (Chan et al., 2021; Mayer et al., 2017). One study reported having a culturally congruent clinician who led MI sessions (Dale, 2020), while “lay counselors” (Psaros et al., 2014) and “research coordinators” (Morris et al., 2022) conducted MI-based sessions in two studies. One study utilized social workers as interventionists (Teixeira da Silva et al., 2021). Moitra et al. (2020) provided no details regarding the credentials, training, or demographic characteristics of the motivational interviewer.

### Duration of Motivational Interview

Three studies reported conducting two MI-based sessions (Chan et al., 2021; Dale, 2020; Moitra et al., 2020), and two reported offering four sessions (Mayer et al., 2017; Teixeira da Silva et al., 2021). Two reported using an individualized approach with a median of 7–10 MI sessions per participant (Morris et al., 2022; Psaros et al., 2014). The duration of MI-based sessions ranged from 15 to 60 min across the studies. In Moitra’s study, the duration of the first MI session ranged from 15 to 25 min, and the median duration of the second session was 5 min (Moitra et al., 2020). Chan et al. (2021) reported 15–20-min sessions, and Mayer et al. reported their MI sessions lasting 50 min (2017). In the Psaros et al. (2014) study, the duration of MI was customized to the individual’s needs, with a median duration of 40 min (IQR 30,50) for the first session and median duration of 20 min (IQR 15, 30) by session 4. There were no reports of MI durations in two of the studies (Dale, 2020; Morris et al., 2022).

### Motivational Interview Content

Most studies reported that the first MI-based session included a combination of providing PrEP education and identifying and alleviating barriers to PrEP use (Chan et al., 2021; Dale, 2020; Mayer et al., 2017; Morris et al., 2022; Psaros et al., 2014; Teixeira da Silva et al., 2021). Chan and colleagues (2021) explicitly mentioned building rapport with participants in the first session. Both the Dale (2020) and Moitra et al. (2020) studies reported 2 sessions, and the second visit reinforced the content presented in the first visit. However, unlike these two studies, Chan et al. (2021) used the second session to develop a PrEP action plan with participants. In one study that utilized 4 MI sessions (Mayer et al., 2017), the authors described how sessions built upon one another and focused on adherence checks, problem solving, risk assessments, and plans for PrEP maintenance. Moitra’s study did not provide any specifications on the MI content in the intervention (Moitra et al., 2020).

### Intervention Effects

One study reported a statistically significant doubling in PrEP appointment attendance for those in the MI-based experimental arm (52.3%) compared to the control group (27.9%) (Chan et al., 2021). Four studies reported a pattern toward improvement in PrEP initiation and/or adherence, but the improvement was not statistically significant (Dale, 2020; Mayer et al., 2017; Moitra et al., 2020; Morris et al., 2022). Analysis of 3-month survey data in one study showed that a significantly greater proportion of participants who received the MI-based intervention initiated PrEP than control group participants (Teixeira da Silva et al., 2021).

### Quality of Evidence

Despite the potential for rigor among the randomized/placebo-controlled trials (Chan et al., 2021; Mayer et al., 2017; Morris et al., 2022; Psaros et al., 2014; Teixeira da Silva et al., 2021), these studies were given “good” quality ratings (Dang & Dearholt, 2018). The randomized trials contained small sample sizes and limited or no information regarding the reliability of measurements, which reduced their ability to identify strong effects and generalizable results. However, they did contain some control with appropriate conclusions and reasonably consistent recommendations given their evidence. The quasi-experimental study by Moitra et al. (2020) was also given a “good” quality rating for similar reasons. The quasi-experimental study conducted by Dale (2020) was rated “low” quality due to a small sample size and reliance on qualitative findings to substantiate claims regarding intervention effects.

## Discussion

This scoping review examined the use and components of MI in interventions aimed at improving PrEP initiation and adherence. Intervention components regarding the duration of MI sessions, MI counselor, and MI content differed across studies. Results from the studies’ small sample sizes and average quality provided inconclusive evidence regarding the relative effects of MI interventions on PrEP initiation and adherence. We were also unable to isolate the effect of MI from other components in the multi-component interventions. Complete details regarding MI components were not provided for all studies. Studies contained important similarities and differences to each other and to traditional MI approaches that require examination and discussion among prevention scientists who develop PrEP interventions and the clinicians and paraprofessionals who implement them.

Effective health promotion interventions are often guided by theories and/or conceptual frameworks whose interrelated concepts explain human behavior (Fishbein & Yzer, 2003; Glanz & Bishop, 2010). All interventions were guided by theoretical frameworks (e.g., social cognitive theory, IMB model), but most lacked clarity regarding how those frameworks guided their MI approach. Considering that MI was originally a standalone communication approach that leveraged client goals as the core intervention for behavior change (Hettema et al., 2005; Rollnick & Miller, 1995), contemporary interventions should explain how MI approaches meld with chosen theoretical constructs to achieve desired health outcomes (in this case PrEP initiation and/or adherence). Since the reviewed studies included multi-component interventions, it is unclear how MI fits into their theoretical frameworks and if the multi-component intervention model still adheres to the “spirit” of MI (Miller & Rollnick, 2012). For example, identifying and resolving ambivalence is key to MI (Miller & Rollnick, 2012; Rollnick & Miller, 1995). However, it is unclear how key constructs of the theoretical approaches used in these studies (e.g., self-efficacy or self-control) support or reinforce behavior change catalyzed by the MI approach in the studies. Additionally, studies in this review lacked key information regarding participants’ goals, their relative initial interests in PrEP initiation and/or adherence, and how theory informed the MI approaches used in session interviews.

Although using a range of trained professionals to conduct MI could be beneficial in clinical and community-based settings in which licensed clinicians are limited, the lack of information about counselors’ demographic characteristics or professional credentialing lessens our ability to make claims regarding interviewer impact on intervention outcomes. Some research suggests that cultural congruence, familiarity, patient-centered communication, and personal disclosures from clinicians improve adherence to clinical recommendations among subpopulations with elevated HIV risk, such as Black SMM (Dangerfield et al., 2021a, b; White et al., 2019). Therefore, we hypothesize that having MI counselors with clinical credentials, standard training in the MI approach, *and* in-group identity markers with potential PrEP clients (e.g., race/ethnicity, gender, and/or sexual or gender identity) could result in better engagement and collaboration, thereby strengthening the effects of MI on behavior change. Since PrEP initiation and adherence are partly impacted by medical mistrust and low perceived HIV risk (Biello et al., 2018; Gallagher et al., 2014), a trained, culturally congruent counselor with personal PrEP use experience could bridge the gap between participants (Dangerfield et al., 2021a). However, the inconsistency of demographic characteristics among the counselors identified in this review limits inferences on PrEP use effects.

Although MI sessions are generally described as brief and a number of sessions vary in health promotion interventions (Hettema et al., 2005; Palacio et al., 2016; Rubak et al., 2005), it is unclear how the range of sessions impacts intervention effects on PrEP initiation and/or adherence among the studies in this review. Potentially, the duration and number of sessions could be key to the relative impact of MI on PrEP use and to standardize this approach in clinical or community-based settings where resources are limited and patient engagement is often challenging (Emmons & Rollnick, 2001). In addition, interventions that effectively increase PrEP initiation and adherence in the shortest amount of time would reduce HIV risk among patients in a timelier manner.

There are limitations to this scoping review. The scope of the review was restricted to peer-reviewed articles printed in English and did not consider studies in other languages. Additionally, this scoping review did not measure the strength of associations between the interventions on PrEP initiation and adherence. Implications for specific populations cannot be drawn. Studies also did not discuss effects on contemporary PrEP modalities such as “on-demand PrEP” and the cabotegravir injectable, both of which could impact willingness, initiation, and adherence (Beymer et al., 2019; Meyers et al., 2018; Molina et al., 2017). Findings from this review will provide a foundation for future research on this important topic, such as conducting meta-analyses.

It was difficult to assess how the MI approaches were designed or how adherent they were to the “spirit of MI” (Miller & Rollnick, 2012) for several interventions. Since contemporary HIV prevention interventions have the ultimate goal of improving PrEP initiation and adherence among vulnerable individuals, the question posed in this scoping review is: are prevention scientists conducting MI or should “MI-consistent” interventions be reclassified as “MI-plus” or something else? Rollnick and Miller (1995, 2009) suggest that MI will not work well if dispatched as a cookbook approach or set of techniques applied *to* clients as opposed to *with* them, which may explain the variation in MI effects because PrEP interventions have an intrinsic goal to encourage initiation and/or adherence. Implementing an intervention to help individuals change unhealthy to healthy behaviors is different than encouraging people to use prophylactic medication for an absent condition because medication non-use may not necessarily be an “unhealthy behavior.” Additionally, encouraging specific behavior change in interventions with desired outcomes in mind (i.e., PrEP initiation and adherence) could deviate from the core element of focusing on client goals and desired behaviors, which could include other HIV prevention behaviors without using PrEP. Therefore, the premise of PrEP interventions that include MI components inherently differs from the “spirit of MI” (Miller & Rollnick, 2009) and should be reconsidered in prevention science.

Based upon the findings of this review, studies that include MI should provide details regarding their intervention components. Specifically, studies should detail (1) the theoretical framework guiding MI-based content and conversations; (2) demographic characteristics and professional qualifications of MI counselors; (3) MI counselor selection process; and (4) justification for location, number, and duration of MI sessions. These guidelines could improve researchers’ ability to standardize MI-based intervention approaches to potentially obtain stronger intervention effects and bring the strategies to scale. Developing a manual for an evidence-based MI intervention is feasible and can facilitate desired behavior change (Hurlocker et al., 2021), furthering the notion that at least some standardization in the MI approach is necessary for PrEP initiation and/or adherence interventions.

## Conclusions

We found variability across studies in this review that makes it difficult to draw inferences about MI implementation in PrEP interventions. In the absence of detailed explanations of the components of MI in PrEP interventions, we are limited in our ability to assess the technique on PrEP outcomes. Substantial limitations regarding small sample sizes, self-reported outcomes, and inconclusive findings hinder our ability to establish standard protocols for MI implementation in PrEP interventions. No study earned an exemplar quality rating. Therefore, none of the studies in this scoping review can serve as a model for how MI can be used effectively in PrEP interventions. Consistent approaches are needed to identify the relative impact of MI on PrEP initiation and adherence to potentially standardize this approach in clinical and community-based settings. Future research should continue to identify the relative impact of MI approaches on PrEP initiation and adherence.
